# Msh2-Msh3 DNA-binding is not sufficient to promote trinucleotide repeat expansions in *Saccharomyces cerevisiae*

**DOI:** 10.1093/genetics/iyae222

**Published:** 2025-01-10

**Authors:** Katherine M Casazza, Gregory M Williams, Lauren Johengen, Gavin Twoey, Jennifer A Surtees

**Affiliations:** Department of Biochemistry, Jacobs School of Medicine and Biomedical Sciences, State University of New York at Buffalo, Buffalo, NY 14203, USA; Department of Biochemistry, Jacobs School of Medicine and Biomedical Sciences, State University of New York at Buffalo, Buffalo, NY 14203, USA; Curia Global, Inc., Buffalo, NY 14203, USA; Department of Biochemistry, Jacobs School of Medicine and Biomedical Sciences, State University of New York at Buffalo, Buffalo, NY 14203, USA; Department of Biochemistry, Jacobs School of Medicine and Biomedical Sciences, State University of New York at Buffalo, Buffalo, NY 14203, USA; Department of Biochemistry, Jacobs School of Medicine and Biomedical Sciences, State University of New York at Buffalo, Buffalo, NY 14203, USA

**Keywords:** mismatch repair, trinucleotide repeat expansions, MSH3, DNA-binding specificity, DNA repair, *Saccharomyces cerevisiae*

## Abstract

Mismatch repair (MMR) is a highly conserved DNA repair pathway that recognizes mispairs that occur spontaneously during DNA replication and coordinates their repair. In *Saccharomyces cerevisiae*, Msh2-Msh3 and Msh2-Msh6 initiate MMR by recognizing and binding insertion or deletion (in/del) loops up to ∼17 nucleotides (nt.) and base–base mispairs, respectively; the 2 complexes have overlapping specificity for small (1–2 nt.) in/dels. The DNA-binding specificity for the 2 complexes resides in their respective mispair binding domains (MBDs) and has distinct DNA-binding modes. Msh2-Msh3 also plays a role in promoting *CAG/CTG* trinucleotide repeat (TNR) expansions, which underlie many neurodegenerative diseases such as Huntington's disease and myotonic dystrophy type 1. Models for Msh2-Msh3's role in promoting TNR tract expansion have invoked its specific DNA-binding activity and predict that the TNR structure alters its DNA binding and downstream activities to block repair. Using a chimeric Msh complex that replaces the MBD of Msh6 with the Msh3 MBD, we demonstrate that Msh2-Msh3 DNA-binding activity is not sufficient to promote TNR expansions. We propose a model for Msh2-Msh3-mediated TNR expansions that requires a fully functional Msh2-Msh3 including DNA binding, coordinated ATP binding, and hydrolysis activities and interactions with Mlh complexes that are analogous to those required for MMR.

## Introduction

Mismatch repair (MMR) is an evolutionarily conserved pathway that recognizes and corrects errors in DNA replication. Two heterodimeric MutS homolog (Msh) complexes initiate MMR through the recognition of distinct DNA structures that arise as a result of nucleotide misincorporation, leading to mispairs, or DNA polymerase slippage events, leading to insertions or deletions (in/dels). Msh2-Msh6, or MutSα, recognizes and binds mispairs and small in/dels [1–2 nucleotides (nt.)] through a conserved Phe-X-Glu motif that intercalates with the mispair, burying it within Msh2-Msh6 (Obmolova *et al*. 2000; Warren *et al*. 2007). Msh2-Msh3, or MutSβ and the focus of this study, recognizes and binds in/dels of up to 17 nucleotides, as well as some mispairs, through a conserved Tyr-Lys pair that interacts with the 5′ double-strand/single-strand DNA junction in loop structures, leaving at least part of the DNA structure accessible (Sia *et al*. 1997; Kunkel and Erie 2005; Lee *et al*. 2007; Dowen *et al*. 2010; Gupta *et al*. 2011).

**Fig. 1.**
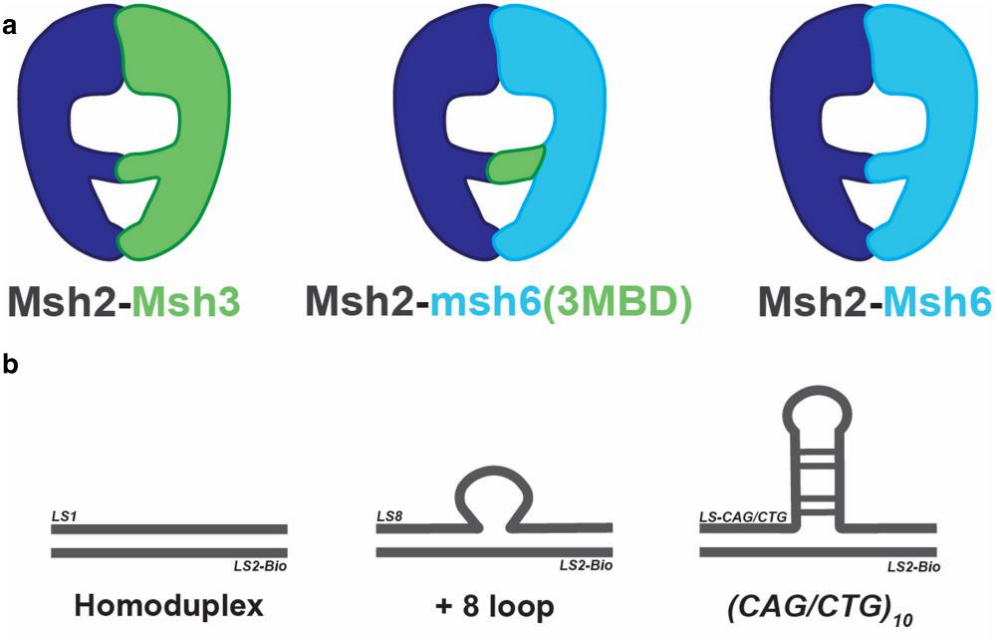
Schematic of MSH complexes and Msh2-Msh3 DNA substrates. a) Schematic indicating the overall structure of the Msh2-Msh3, Msh2-Msh6, and chimeric MSH complexes. Msh2msh6(3MBD) results from replacing the MBD of Msh6 with that of Msh3, which is sufficient to switch DNA-binding specificities and DNA hopping capacity (Shell *et al*. 2007; Brown *et al*. 2016). b) Schematic depicting the DNA structures used in this study: nonspecific homoduplex DNA, an MMR-specific loop structure with 8 extrahelical nucleotides [(GT)_4_] and TNR tract slipped structures for *CAG* and *CTG* tracts. The DNA sequences are in Table 1.

The mispair binding domains (MBDs) of Msh3 and Msh6 are responsible for Msh2-Msh3 vs Msh2-Msh6 structure-specific DNA-binding activities and contain the highly conserved Phe-X-Glu (Msh6) or Tyr-Lys (Msh3) motifs, as well as other highly conserved residues that contribute to DNA structure specificity (Obmolova *et al*. 2000; Warren *et al*. 2007; Gupta *et al*. 2011). Replacing the Msh6 MBD with the Msh3 MBD in the context of Msh2-Msh6 (Fig. 1) swapped the structure-binding specificity of the resulting Msh2-msh6(3MBD) complex, which exhibited a preference for Msh2-Msh3 in/del loop (IDL) substrates (Shell *et al*. 2007; Brown *et al*. 2016). The ATPase activity of Msh2-msh6(3MBD) was stimulated by Msh2-Msh3 substrates, and this chimeric complex gained Msh2-Msh3's ability to bypass protein blocks, “hopping” over nucleosomes (Brown *et al*. 2016), which Msh2-Msh6 lacks (Gorman *et al*. 2007; Brown *et al*. 2016). These data indicate that Msh2-Msh3's DNA-binding specificity is largely mediated through its MBD.

Once bound, Msh complexes bind ATP and recruit MutL homolog (Mlh) heterodimeric complexes MutLα and/or MutLγ in an ATP-dependent manner. The latent endonuclease activity of the Mlh complexes is activated by Msh and PCNA, nicking the nascent DNA strand (Furman *et al*. 2021; Pannafino and Alani 2021). This is followed by the recruitment of Exo1 and other downstream factors that promote the excision and resynthesis of the nascent DNA to complete repair (Jiricny 2006; Li 2008; Goellner *et al*. 2015; Keogh *et al*. 2017). ATP hydrolysis by Msh2-Msh3 is thought to be important for the turnover of the complex, allowing it to rebind DNA (Kijas *et al*. 2003; Owen *et al*. 2005, 2009; Kumar *et al*. 2014).

Loss of *MSH3* compromises genome stability and leads to an increase in microsatellite instability (Marsischky *et al*. 1996; Sia *et al*. 1997; Harrington and Kolodner 2007; Lee *et al*. 2007; Kumar *et al*. 2013). Single nucleotide polymorphisms in human *MSH3* have been associated with a predisposition to cancer (Miao *et al*. 2015; De’ Angelis *et al*. 2018; Santos *et al*. 2018; Aelvoet *et al*. 2023; Rashid *et al*. 2024). These genome-protective characteristics of *MSH3* are in direct contrast to its pathogenic role in promoting trinucleotide repeat (TNR) expansions (Kantartzis *et al*. 2012; Williams and Surtees 2015; Keogh *et al*. 2017). TNR expansions are the cause of over 40 neurodegenerative and neuromuscular diseases such as Huntington's disease and myotonic dystrophy type 1, which are caused by *CAG* and *CTG* expansions, respectively (Mirkin 2007). *MSH3* promotes TNR expansions, including *CNG* tracts, in multiple model systems including yeast, mice, and human cell culture (Kantartzis *et al*. 2012; Lahue 2020; Iyer and Pluciennik 2021; Richard 2021; Matos-Rodrigues *et al*. 2023). In genome-wide association studies, *Msh3* was identified as a genetic modifier of TNR expansions; polymorphisms identified in *Msh3* that correlated with higher *Msh3* expression exhibited increased TNR tract instability in mice (Tomé *et al*. 2013; Bettencourt *et al*. 2016; Lee *et al*. 2019). In contrast, *MSH6* does not promote significant *CNG* expansions (Kantartzis *et al*. 2012; Iyer and Pluciennik 2021), indicating that MMR-dependent TNR expansions are specific to the Msh2-Msh3-mediated pathway, leading us to focus on the molecular activities of Msh2-Msh3 that are necessary for promoting TNR expansion.

Msh2-Msh3 recognizes and binds the slipped strand secondary structures thought to form during replication of TNR tracts, with affinities similar to in/dels (Owen *et al*. 2005; Tian *et al*. 2009; Lang *et al*. 2011). However, the execution of repair is altered, leading to TNR tract expansions rather than repair pathways that maintain tract length. While Msh2-Msh3 ATPase activity is likely required for TNR expansions (Tomé *et al*. 2009; Keogh *et al*. 2017), TNR DNA structures decreased Msh2-Msh3 ATP binding and hydrolysis activities compared to that observed with an in/del MMR substrate (Owen *et al*. 2005, 2009). Similarly, Msh2-Msh3 nucleotide-binding and hydrolysis activities are differentially modified in the presence of DNA structures it binds in double-strand break repair vs MMR (Kumar *et al*. 2014). These observations led to the prediction that Msh2-Msh3 becomes “trapped” upon binding TNR structures, preventing the repair of these slipped strand secondary structures (Owen *et al*. 2005; Lang *et al*. 2011). Our in vitro work has suggested that at least part of Msh2-Msh3's role in promoting TNR expansions is in stabilizing the TNR structures (Kantartzis *et al*. 2012), consistent with other studies (Tian *et al*. 2009). In this study, we sought to determine whether Msh2-Msh3's specific DNA-binding activity, through the Msh3 MBD, is sufficient to promote TNR expansions.

## Materials and methods

### Protein purification

Msh2-Msh3 was overexpressed in *Saccharomyces cerevisiae* and purified as previously described (Kumar *et al*. 2014). Msh2-msh6(3MBD) and Msh2-Msh6 were overexpressed in *Escherichia coli* and purified as previously described (Brown *et al*. 2016).

### DNA substrates

DNA substrates were constructed with synthetic oligonucleotides (Table 1). One oligonucleotide in each substrate was biotinylated for attachment to streptavidin tips (see below). Oligonucleotides were mixed at equimolar concentrations in 100 mM NaCl, 10 mM MgCl_2_, and 0.1 mM EDTA, heated to 95°C for 5 minutes and allowed to cool slowly to room temperature.

**Table 1.** DNA substrates.

DNA structure	Top strand (5′–3′)	Bottom strand (5′–3′) (LS2-Bio)
Homoduplex	cacgctaccgaattctgacttgctaggcatctttgcccacgttgaccc (LS1)	Bio/gggtcaacgtgggcaaagatgtcctagcaagtcag aattcggtagcgtg
+8 loop	cacgctaccgaattctgacttgctaggtgtgtgtgacatctttgccca cgttgaccc (LS8)	Bio/gggtcaacgtgggcaaagatgtcctagcaagtcag aattcggtagcgtg
(*CAG*)_10_	cacgctaccgaattctgacttgctagcagcagcagcagcagcagc agcagcagcaggacatctttgcccacgttgaccc (LS-CAG)	Bio/gggtcaacgtgggcaaagatgtcctagcaagtcag aattcggtagcgtg
(*CTG*)_10_	cacgctaccgaattctgacttgctagctgctgctgctgctgctgctgct gctgctggacatctttgcccacgttgaccc (LS-CTG)	Bio/gggtcaacgtgggcaaagatgtcctagcaagtcag aattcggtagcgtg

### Biolayer interferometry

Biolayer interferometry (BLI) experiments were performed with purified Msh2-Msh3, Msh2-Msh6, and Msh2-msh6(3MBD) using a Sartorius Octet Red96e. DNA concentration was optimized for each protein complex in order to have ideal ligand density across the streptavidin sensor for each protein complex. A titration of DNA concentrations was loaded onto the sensor. Protein association was measured for each protein at a concentration 10–20 times higher than its predicted K_D_, (Surtees and Alani 2006; Shell *et al*. 2007; Kumar *et al*. 2011; Brown *et al*. 2016). The ligand (DNA) concentration chosen was the lowest DNA concentration that exhibited strong binding signal without reaching saturation and fit well to a 1:1 model. The DNA substrate concentration selected (7.5 nM) was the same for Msh2-Msh3 and Msh2-mh6(3MBD). The Msh2-Msh6 experiments required a higher DNA concentration (30 nM); the association curves for Msh2-Msh6 displayed weak binding signal at 7.5 nM, which is consistent with improper ligand density, as recommended by the Sartorius Octet application manual.

BLI experiments were performed in binding buffer (25 mM Tris-HCl pH 7.5, 1 mM EDTA, 100 mM NaCl, 0.1 mg/mL BSA, 1 mM DTT, and 0.05% Tween-20) using the Octet Red96e system (ForteBio). Biotinylated DNA substrates were immobilized to prehydrated streptavidin biosensors. Biosensors were then incubated with 50 uM biocytin in SuperBlock buffer (Thermo Fisher) to quench unbound streptavidin and equilibrated in binding buffer. Biosensors were incubated with Msh2-Msh3 or Msh2-msh6(3MBD) (250, 125, 62.5, 31.25, 15.6, and 7.8 nM) or with Msh2-Msh6 (150, 75, 37.5, 18.75, 9.4, and 4.7 nM) for 180 s. Biosensors were then moved to wells containing binding buffer to allow for dissociation for 180 s. Data were collected using Data Acquisition 12.0 software (ForteBio) and analyzed with Data Analysis HT 12.0 software (ForteBio). Data were fit to 1:1 model to obtain binding kinetics. Experiments were performed in triplicate with protein obtained from at least 2 independent purifications.

### Strains and Media

All strains and plasmids used are listed in Tables 2 and 3. All yeast transformations were performed using the lithium acetate method (Gietz *et al*. 1992). Yeast strains were derived from the S288c background: FY23 for slippage assays and FY86 for TNR expansion assays (Winston *et al*. 1995). *msh3Δ* and *msh6Δ* were constructed by amplifying a chromosomal mutant::KANMX fragment from the yeast deletion collection that was integrated into the respective chromosomal location. *msh6(3MBD)* was integrated into the endogenous MSH6 locus in a *msh3Δ* strain using pRDK4576 as described previously (Shell *et al*. 2007).

**Table 2.** Strains used in this study.

JSY#	Other name	Relevant genotype	Strain background	Source
126	FY23	*MATa ura3-52 leu2Δ1 trp1Δ63 his3Δ200 lys2Δ202 ura3Δ leu2Δ trp1Δ*		Winston *et al*. (1995)
127	FY86	*MATα ura3-52 leu2Δ1 trp1Δ63 his3Δ200 lys2Δ202 ura3Δ leu2Δ his3Δ*		Winston *et al*. (1995)
485		*msh6Δ::KANMX*	FY86	Kumar *et al*. (2011)
1472		*msh3Δ::KANMX*	FY86	Kantartzis *et al*. (2012)
314	EAY337	*msh6::hisG*	FY23	Bowers *et al*. (1999)
905	EAY420	*msh3Δ::hisG*	FY23	Studamire *et al*. (1998)
3925, 3926, 3927		*msh3Δ::hisG msh6(3MBD)*	FY23	This study
3554, 3555, 3556		*msh3Δ::hisG msh6(3MBD*)	FY86	This study

**Table 3.** Plasmids used in this study.

JSB#	Plasmid name	Plasmid description	Source
94	pBK1	(CAGT)_16_-*URA3* slippage assay reporter	Sia *et al*. (1997)
95	pMD28	(G)_18_-*URA3* slippage assay reporter	Sia *et al*. (1997)
299	pSH44	(GT)_16.5_-*URA3* slippage assay reporter	Henderson and Petes (1992)
361	pBL139	*(C,A,G)_25_::URA3::HIS3* integration plasmid	Miret *et al*. (1998)
363	pBL69	*(CTG)_25_::URA3::HIS3* integration plasmid	Miret *et al*. (1998)
364	pBL70	*(CAG)_25_::URA3::HIS3* integration plasmid	Miret *et al*. (1998)
365	pBL138	*(C,T,G)_25_::URA3::HIS3* integration plasmid	Miret *et al*. (1998)

TNR substrates were integrated into all strains as described previously (Dixon *et al*. 2004; Williams and Surtees 2018): (1) (CAG)_25_ (pBL70), (2) (CTG)_25_ (pBL69), (3) scrambled (C,A,G)_25_ (pBL139), and (4) scrambled (C,T,G)_25_ (pBL138) (Table 3) (Dixon *et al*. 2004). The repeat tracts were in the promoter of the *URA3* reporter gene; expansions of ≥4 repeats alter the transcriptional start site, making the cells resistant to 5-FOA.

The microsatellite instability constructs have been described previously (Table 3) (Sia *et al*. 1997). Repeat tracts of 1 nt. [(G)_18_], 2 nt. [(GT)_16.5_], or 4 nt. [(CAGT)_16_] were placed in-frame upstream of *URA3*. Unrepaired slippage events shift *URA3* out of frame, resulting in 5-FOA resistance.

### Western blot

We performed western blots of titrations of purified Msh2-Msh6 and Msh2-msh6(3MBD) to test the sensitivity of the anti-Msh6 antibody. Titrations of purified protein were loaded onto a 6% SDS-PAGE gel. The gel was transferred to nitrocellulose using the Bio-Rad Trans-Blot Turbo Transfer system. The blot was blocked overnight at 4°C and then incubated for 2 h in 1:2,000 dilution of anti-Msh6 primary rabbit antibody (Kumar *et al*. 2011). It was then incubated for 1 h in 1:4,000 dilution of HRP-conjugated anti-rabbit secondary goat antibody. The blot was then imaged using SuperSignal West Atto Ultimate Sensitivity ECL substrate (Thermo Fisher) and imaged using ChemiDoc MP imaging system (Bio-Rad).

To compare endogenous Msh6 and msh6(3MBD) levels, wild type (FY23 and FY86), *msh6(3MBD) msh3Δ* (in the FY23 and FY86 background), and *msh6Δ* (FY23 background) were grown in 1L YPD cultures until OD_600_ = 0.6. Cells were harvested and resuspended in MSH buffer [25 mM Tris–HCl (pH 7.5), 1 mM EDTA, and 175 mM NaCl]. The resuspension was then frozen by dropping into liquid nitrogen, and the frozen cell resuspension was ground with dry ice to lyse. The lysate was then thawed and β-mercaptoethanol and PMSF were added to final concentrations of 10 and 1 mM, respectively. The resuspension was then centrifuged and the cleared lysate was retained. The cleared lysate was concentrated using Amicon Ultra Centrifugal Filter Unit 100 kDa cutoff. Total protein concentration was measured by Bradford assay.

Four hundred micrograms of each lysate as well as *msh6Δ* lysate +0.15 μg of purified Msh2-Msh6 were incubated with anti-Msh6 primary rabbit antibody at 4°C for 1 h while rocking. Fifty microliters of 50% slurry Protein A/G-Agarose beads (Pierce) were added to each and incubated overnight at 4°C while rocking. The beads were incubated on ice for 10 min and then collected by centrifugation and the supernatant was removed. The beads were washed 3 times with IP buffer (50 mM HEPES, pH 7.5, 1 mM EDTA, and 1% Triton-X). Beads were resuspended in 1× Laemmli buffer and heated for 10 min at 95°C. The immunoprecipitated product was resolved in a 4–20% gradient gel (Bio-Rad) and transferred to nitrocellulose using the Bio-Rad Trans-blot Turbo Transfer system. The blot was blocked for 1 h at 4°C and then incubated overnight in 1:1,000 dilution of anti-Msh6 primary rabbit antibody. It was then incubated for 1 h in 1:4,000 dilution of HRP-conjugated anti-rabbit secondary goat antibody. The blot was then imaged using SuperSignal West Atto Ultimate Sensitivity ECL substrate (Thermo Fisher) and imaged using ChemiDoc MP imaging system (Bio-Rad).

### Microsatellite instability assay

Microsatellite instability assays were performed as described (Sia *et al*. 1997). Single colonies were grown on SC-tryptophan (SC-trp) to maintain the reporter plasmids. Colonies of ∼2 mm were selected from ≥3 independent isolates of each genetic background. Individual colonies were resuspended in 3 mL of liquid SC-trp and incubated with shaking for 20 h at 30°C. Overnight cultures were serial diluted and plated on permissive (SC-trp) and selective (SC-trp +5-FOA) plates. Plates were incubated at 30°C for 2–4 days. Mutation rates were calculated by the method of the median (Drake 1991).

95% confidence intervals were determined using tables of confidence intervals for the median (Nair 1940; Dixon and Massey 1969). *P*-values were determined by Mann–Whitney rank analysis in GraphPad Prism.

### Assay for TNR expansion

TNR expansion assays were performed as described (Williams and Surtees 2018). Briefly, single colonies were obtained on synthetic medium (SC) lacking histidine (SC-his) or lacking histidine and uracil (SC-his-ura) for *msh6(3MBD) msh3Δ*. Individual colonies were selected from ≥3 independent isolates of each genetic background and were assayed. Colonies with unexpanded tracts, which were confirmed by PCR analysis, were diluted and plated on SC-his and incubated at 30˚C for 3–4 days to allow expansions to occur. Several ∼2 mm colonies were selected, diluted and plated onto permissive (SC-his) and selective (SC-his +5-FOA), and incubated at 30°C from 3–4 days. Colonies were counted and expansion rates were calculated as described (Drake 1991). The 95% confidence intervals were determined from tables of confidence intervals for the median (Nair 1940; Dixon and Massey 1969). *P*-values were determined by Mann–Whitney rank analysis (Nair 1940; Dixon and Massey 1969; Drake 1991; Sia *et al*. 1997) in GraphPad Prism.

True expansions were determined as previously described (Williams and Surtees 2015, 2018; Williams *et al*. 2020), by amplifying the reporter promoter region with SO295 (AAACTCGGTTTGACGCCTCCCATG) and SO296 (AGCAACAGGACTAGGATGAGTAGC) and digesting with *SphI* to release the TNR tract. Tract mobility was assessed by electrophoresis through a 12% native polyacrylamide gel (0.5× TBE). At least 30 independent 5-FOA-resistant colonies were characterized for each tract and genotype combination. Sequencing of the *URA3* gene was performed to identify mutations in *URA3* that may lead to 5-FOA resistance. The *URA3* coding region was amplified from genomic preps derived from 5-FOA-resistant colonies that did not exhibit expanded TNR tracts, using SO295 and SO1079 (GTTAGAAGTGCGGTTGATGTCG). This amplicon included the *URA3* and TNR tract sequences, to confirm the tract size in conjunction with the *URA3* sequence. We also amplified the promoter region of this reporter construct using SO296 and SO1080 (GGGAACAAAAGCTGGTACCGGG). The amplified regions were sent for Oxford Nanopore sequencing (Plasmidsaurus).

## Results

### Msh2-Msh3 and Msh2-msh6(3MBD) exhibit specific binding to TNR structures in vitro

Previous studies, including our own, have indicated that Msh2-Msh3 TNR slipped strand structure binding and stabilization is required to promote TNR expansions (Owen *et al*. 2005; Panigrahi *et al*. 2005; Kantartzis *et al*. 2012; Williams and Surtees 2015). In this study, we set out to test whether the structure-specific binding activity of Msh2-Msh3 is *sufficient* to promote TNR expansions, using the msh6(3MBD) chimeric protein (Fig. 1). Before testing Msh2-msh6(3MBD) activities in vivo, we characterized its in vitro DNA-binding properties, using both MMR and TNR DNA substrates (Fig. 1) (Surtees and Alani 2006). We previously demonstrated yeast Msh2-Msh3 specificity for +8 loop DNA structures, substrates for Msh2-Msh3-mediated MMR, using electrophoretic mobility assays (EMSAs) and DNA footprinting (Surtees and Alani 2006; Lee *et al*. 2007; Brown *et al*. 2016). Human Msh2-Msh3 was similarly demonstrated to have specificity for DNA loop structures (Habraken *et al*. 1996; Wilson *et al*. 1999; Owen *et al*. 2005, 2009; Lang *et al*. 2011). Here, we used BLI to characterize yeast Msh2-Msh3 and Msh2-msh6(3MBD) binding to homoduplex DNA, a (GT)_4_ loop (+8 loop) (Surtees and Alani 2006), (*CAG*)_10_ or (*CTG*)_10_ hairpin DNA structures, using biotinylated DNA structures assembled from synthetic oligonucleotides (Surtees and Alani 2006).

Msh2-Msh3 exhibited significant nonspecific binding activity to homoduplex and exhibited ∼3- to 4-fold increased affinity for the +8 loop (Table 4; K_D_). The relative affinities are consistent with our previous estimates using EMSA, although the current K_D_'s are ∼20-fold lower (Surtees and Alani 2006). The association rate (Table 4; k_a_) was similar for both substrates; the dissociation rate with the +8 loop (Table 4; k_dis_) was slower. Msh2-Msh3 also bound preferentially to *(CTG)_10_* or *(CAG)_10_* quasi-hairpin structures with affinities ∼4- and 7-fold higher than to homoduplex, respectively. This is consistent with human Msh2-Msh3, which exhibited binding to *CAG* tracts similar to +8 loop structures (Owen *et al*. 2005), although we note that the affinities differ. The higher affinities of Msh2-Msh3 to all 3 DNA structures are driven primarily by decreased k_dis_, although there was also an ∼2-fold increase in k_a_ with specific DNA substrates.

**Table 4.** Binding kinetics of Msh2-Msh3 and Msh2-msh6(3MBD).

Msh2-Msh3						
Substrate	K_D_ (M)	K_D_ error	k_a_ (1/Ms)	k_a_ error	k_dis_ (1/s)	k_dis_ error
Homoduplex	9.22 × 10^−9^	1.42 × 10^−10^	3.33 × 10^5^	2.20 × 10^3^	3.07 × 10^−3^	4.26 × 10^−5^
+8 Loop	2.62 × 10^−9^	5.28 × 10^−11^	6.57 × 10^5^	3.46 × 10^3^	1.72 × 10^−3^	3.35 × 10^−5^
(*CAG*)_10_	1.38 × 10^−9^	4.84 × 10^−11^	8.69 × 10^5^	5.63 × 10^3^	1.20 × 10^−3^	4.13 × 10^−5^
(*CTG*)_10_	2.25 × 10^−9^	4.87 × 10^−11^	6.96 × 10^5^	3.68 × 10^3^	1.57 × 10^−3^	3.29 × 10^−5^
**Msh2-msh6(3MBD)**						
**Substrate**	**K_D_ (M)**	**K_D_ error**	**k_a_ (1/Ms)**	**k_a_ error**	**k_dis_ (1/s)**	**k_dis_ error**
Homoduplex	7.16 × 10^−9^	6.81 × 10^−11^	1.80 × 10^6^	1.51 × 10^4^	1.29 × 10^−2^	5.83 × 10^−5^
+8 Loop	8.72 × 10^−10^	9.52 × 10^−12^	2.32 × 10^6^	1.01 × 10^4^	2.03 × 10^−3^	2.03 × 10^−5^
(*CAG*)_10_	2.37 × 10^−9^	2.02 × 10^−11^	2.58 × 10^6^	1.53 × 10^4^	6.10 × 10^−3^	3.72 × 10^−5^
(*CTG*)_10_	1.05 × 10^−9^	1.42 × 10^−11^	1.94 × 10^6^	8.34 × 10^3^	2.03 × 10^−3^	2.60 × 10^−5^

We next tested the DNA-binding kinetics of Msh2-msh6(3MBD), which replaces the Msh6 MBD with Msh3 MBD within the context of Msh2-Msh6 (Fig. 1) (Surtees and Alani 2006; Shell *et al*. 2007; Brown *et al*. 2016). Msh2-msh6(3MBD) displayed a higher (∼8-fold) affinity (Table 4; K_D_) for the +8 loop structure compared to the homoduplex DNA structure, consistent with previous observations (Shell *et al*. 2007; Brown *et al*. 2016) and with Msh2-Msh3 binding (Table 4) (Surtees and Alani 2006). Msh2-msh6(3MBD) also exhibited increased affinity for both TNR hairpin structures, relative to homoduplex, with affinities similar to its affinity for the +8 loop substrate (Table 4; K_D_), as observed with Msh2-Msh3 (Table 4). Msh2-msh6(3MBD) exhibited similar k_a_'s for all of the substrates, which were somewhat higher than Msh2-Msh3 k_a_'s for the same substrates. As with Msh2-Msh3, the increased affinity to specific DNA structures was largely driven by decreased dissociation rates (Table 4; k_dis_). These data indicate that Msh2-Msh3 and Msh2-msh6(3MBD) exhibit similar DNA-binding affinities and that Msh2-msh6(3MBD) binds TNR structures with an affinity similar to its affinity for MMR (+8 loop) structures and is therefore competent to recognize these structures, should they form in vivo.

We also examined binding kinetics of Msh2-Msh6 to the same DNA structures. Msh2-Msh6 displays distinct binding behavior compared to Msh2-Msh3 and Msh2-msh6(3MBD) (Shell *et al*. 2007; Brown *et al*. 2016), requiring reoptimization of BLI conditions to measure the binding kinetics. Msh2-Msh6 displayed a ∼2- to 3-fold higher K_D_ with homoduplex (Table 5), compared to Msh2-Msh3 and Msh2-msh6(3MBD). Msh2-Msh6 exhibited a modest (2-fold) preference for the +8 loop compared to the homoduplex. We have previously shown that this interaction is significantly less stable than Msh2-Msh6 binding to a mispair or +1 insertion (Jiang *et al*. 2005; Brown *et al*. 2016). Interestingly, we observed more significantly increased affinity of Msh2-Msh6 for TNR structures, ∼7-fold over homoduplex. Therefore, Msh2-Msh3, Msh2-msh6(3MBD), and Msh2-Msh6 exhibit similar specificity for the TNR quasi-hairpin. However, *MSH6* does not promote TNR expansions (Kantartzis *et al*. 2012), indicating that TNR binding is not sufficient for promoting expansions.

**Table 5.** Binding kinetics of Msh2-Msh6.

Msh2-Msh6						
Substrate	K_D_ (M)	K_D_ error	k_a_ (1/Ms)	k_a_ error	k_dis_ (1/s)	k_dis_ error
Homoduplex	2.10 × 10^−8^	2.62 × 10^−10^	2.63 × 10^5^	2.15 × 10^3^	5.54 × 10^−3^	5.21 × 10^−5^
+8 Loop	8.67 × 10^−9^	1.27 × 10^−10^	4.55 × 10^5^	3.35 × 10^3^	3.95 × 10^−3^	5.00 × 10^−5^
(*CAG*)_10_	2.73 × 10^−9^	1.82 × 10^−11^	2.22 × 10^6^	1.01 × 10^4^	6.06 × 10^−3^	2.96 × 10^−5^
(*CTG*)_10_	3.07 × 10^−9^	2.58 × 10^−11^	1.32 × 10^6^	5.93 × 10^3^	4.06 × 10^−3^	2.89 × 10^−5^

### *Msh6(3MBD)* retains some Msh2-Msh3-specific MMR function

**Fig. 2.**
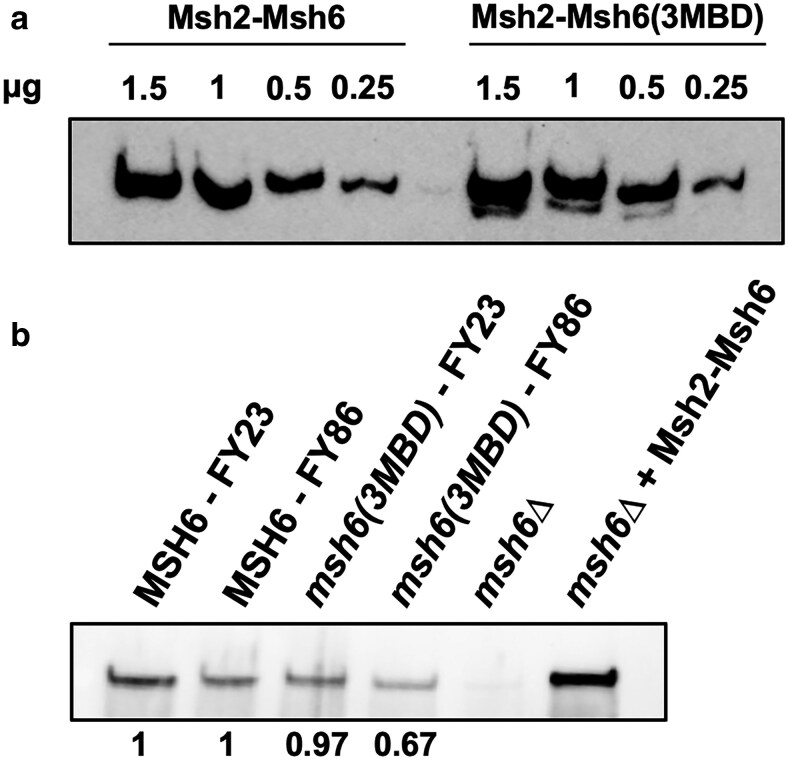
Western blot detecting endogenous Msh6 and Msh6(3MBD). a) Western blot of titration of purified Msh2-Msh6 and Msh2-msh6(3MBD). b) Western blot of immunoprecipitated endogenous Msh6 and msh6(3MBD). Purified Msh2-Msh6 (0.15 μg) was added to *msh6Δ* lysate as a positive control. Msh6/msh6(3MBD) bands were quantified and normalized to a background band (not shown). Normalized relative values of msh6(3MBD) were calculated and compared to Msh6 in their respective backgrounds and are shown below each lane.

To directly test its activity to promote Msh2-Msh3-mediated activities in vivo, we integrated the *msh6(msh3MBD)* construct (Shell *et al*. 2007) into the chromosome, replacing endogenous *MSH6*, in a *msh3Δ* background. This results in a single MSH complex in vivo, Msh2-msh6(3MBD) (Fig. 1). Previous work has demonstrated that *msh6(msh3MBD) msh3Δ* has in vivo MMR activity, indicating that the complex is functional, although both Msh2-Msh6- and Msh2-Msh3-mediated MMRs were compromised to some extent (Shell *et al*. 2007). We performed western blots to compare Msh6 and msh6(3MBD) protein levels in these strains in vivo. We first tested the sensitivity of our polyclonal anti-Msh6 antibody (Kumar *et al*. 2011) for Msh6 compared to msh6(3MBD), using purified proteins. The antibody recognized both proteins with comparable sensitivity (Fig. 2a). From cleared cell lysates, we immunoprecipitated and detected both Msh6 and msh6(3MBD) (Fig. 2b). Levels of Msh6 and msh6(3MBD) were normalized to a background band and quantified to compare protein levels in FY23 and FY86, the backgrounds used for slippage assays and TNR expansions assays, respectively. msh6(3MBD) levels were comparable to Msh6, although it was slightly reduced, ∼70% of Msh6 in the FY86 background. These data indicate that the chimeric complex is stable in vivo (Fig. 2b).

**Fig. 3.**
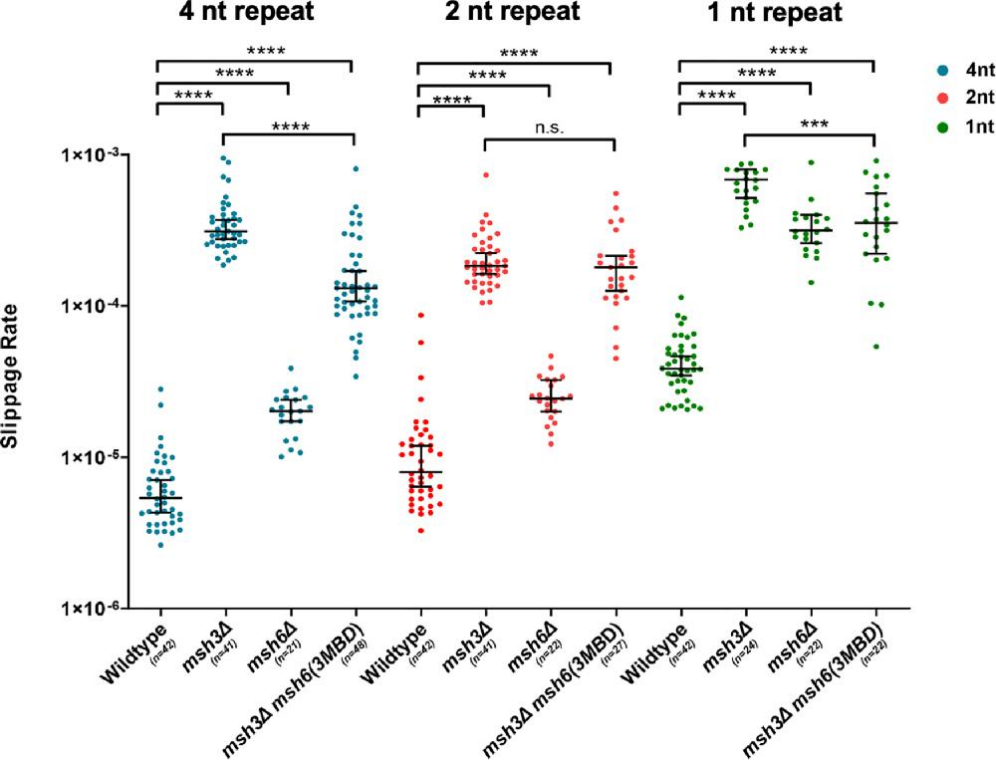
Mutation rate in a slippage mutation rate assay. The rate of slippage events, which push *URA3* out of frame and allow selection in the presence of 5-FOA, was determined in different genetic backgrounds, as indicated. Slippage rate in the presence of mononucleotide (right panel; green), dinucleotide (middle panel; red), and tetranucleotide (left panel; blue) repeats was determined. Median rates (95% confidence interval) are as follows: 4 nt. (blue): wild type: 5.38 × 10^−6^ (4.30 × 10^−6^ to 7.08 × 10^−6^), *msh3Δ*: 3.12 × 10^−4^ (2.77 × 10^−4^ to 3.71 × 10^−4^), *msh6Δ*: 2.01 × 10^−5^ (1.73 × 10^−5^ to 2.4 × 10^−5^), and *msh6(3MBD) msh3Δ*: 1.29 × 10^−4^ (1.07 × 10^−4^ to 1.70 × 10^−4^); 2 nt. (red): wild type: 7.99 × 10^−6^ (6.14 × 10^−6^ to 1.20 × 10^−5^), *msh3Δ*: 1.84 × 10^−4^ (1.63 × 10^−4^ to 2.23 × 10^−4^), *msh6Δ*: 2.45 × 10^−5^ (2.01 × 10^−5^ to 3.24 × 10^−5^), and *msh6(3MBD) msh3Δ*: 1.80 × 10^−4^ (1.26 × 10^−4^ to 2.14 × 10^−4^); and 1 nt. (green): wild type: 3.84 × 10^−5^ (3.47 × 10^−5^ to 4.64 × 10^−5^), *msh3Δ*: 6.85 × 10^−4^ (5.18 × 10^−4^ to 8.02 × 10^−4^), *msh6Δ*: 3.17 × 10^−4^ (2.61 × 10^−4^ to 4.00 × 10^−4^), and *msh6(3MBD) msh3Δ*: 3.53 × 10^−4^ (2.21 × 10^−4^ to 5.55 × 10^−4^). Error bars indicate 95% confidence intervals. *P*-values were calculated using Mann–Whitney test: ****P < 0.0001; ***0.0001 < *P* < 0.001.

*msh6(msh3MBD) msh3Δ* function was previously tested with reporter assays that select for −1 frameshift deletion or +2 frameshift insertions (Shell *et al*. 2007), substrates for both Msh2-Msh3 and Msh2-Msh6 (Marsischky *et al*. 1996; Sia *et al*. 1997). *msh6(3MBD) msh3Δ* elevated mutation rates (30- to 70-fold increases), but not the synergistic >1,000-fold increase observed in *msh3Δ msh6Δ* (Shell *et al*. 2007), indicating that *msh6(3MBD)* retained significant MMR function. Here, we tested whether the chimeric protein would act in repair of larger IDLs that is dependent almost exclusively on Msh2-Msh3-mediated repair (Sia *et al*. 1997; Lee *et al*. 2007; Kumar *et al*. 2013). We used a microsatellite instability reporter plasmid that places a tetranucleotide (*CAGT*)_16_ repeat in-frame upstream of *URA3* (Sia *et al*. 1997). Unrepaired DNA slippage events alter the *URA3* reading frame, resulting in 5-FOA resistance, which allows the selection of these slippage events. Previous work demonstrated that these slippage events result primarily in deletions (Sia *et al*. 1997; Lamb *et al*. 2022). Compared to wild type, *msh3Δ* increased the 4 nt. slippage rate by 58-fold; *msh6Δ* exhibited only a 3-fold increase, consistent with its limited role in repair of these longer in/dels (Fig. 3; blue circles). *msh2Δ* exhibited a slippage rate similar to *msh3Δ* with this 4 nt. repeat (57- and 62-fold increases over wild type, respectively) (Sia *et al*. 1997; Lee *et al*. 2007), consistent with *MSH6* playing only a minor role in repair of larger IDL repair*. msh6(3MBD) msh3Δ* partially complemented the *msh3Δ*, with an intermediate slippage rate, with a 24-fold increase (Fig. 3). This incomplete complementation is consistent with distinct repair mechanisms for Msh2-Msh3 vs Msh2-Msh6 but is consistent with *msh6(3MBD*) allowing recognition of IDL substrates in vivo. We also tested *msh6(3MBD) msh3Δ* function in repair of 1 nt [(G)_18_] and 2 nt [(GT)_16.5_] repeats (Sia *et al*. 1997; Lee *et al*. 2007). *msh6(3MBD) msh3Δ* partially complemented repair of 1 nt. slippage events, compared to *msh3Δ*, but not repair of 2 nt. slippage events. We note that *msh2Δ* exhibited significantly higher slippage rates than either *msh3Δ* or *msh6Δ* or *msh6(3MBD*) in the presence of 1 nt. and 2 nt. repeat reporters (Sia *et al*. 1997; Lee *et al*. 2007), consistent with partial function of *msh6(3MBD)* in repair of these repeats (Fig. 3) (Shell *et al*. 2007).

### *Msh6(3MBD)* does not promote TNR expansions in vivo

**Fig. 4.**
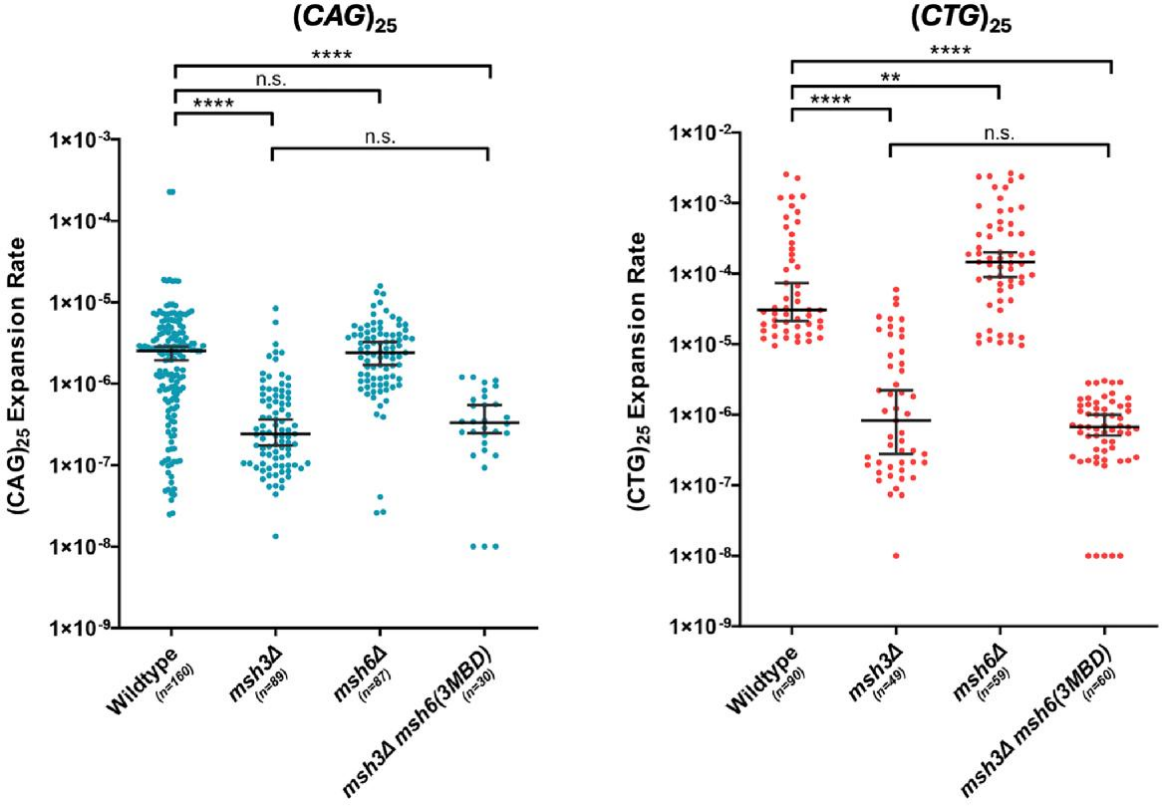
TNR expansion rates in vivo. The rate of expansion events, which prevent expression of *URA3* and allow selection in the presence of 5-FOA, was determined in different genetic backgrounds, as indicated. Expansion rates of (*CAG*)_25_ tracts (left; blue) and (*CTG*)_25_ tracts (right; red) were measured. Error bars indicate 95% confidence intervals. Median rates (95% confidence interval) are as follows: (*CAG*)_25_ (blue): wild type: 1.1 × 10^−6^ (7.0 × 10^−7^ to 1.9 × 10^−6^), *msh3Δ*: 2.4 × 10^−7^ (1.6 × 10^−7^ to 2.8 × 10^−7^), *msh6Δ*: 2.4 × 10^−6^ (1.7 × 10^−6^ to 2.9 × 10^−6^), and *msh6(3MBD) msh3Δ*: 2.5 × 10^−7^ (1.3 × 10^−7^ to 3.4 × 10^−7^); and (CTG)_25_ (red): wild type: 2.2 × 10^−5^ (1.7 × 10^−5^ to 2.9 × 10^−5^), *msh3Δ*: 1.0 × 10^−6^ (2.8 × 10^−7^ to 2.2 × 10^−6^), *msh6Δ*: 1.5 × 10^−4^ (9.0 × 10^−5^ to 2.0 × 10^−4^), and *msh6(3MBD) msh3Δ*: 6.6 × 10^−7^ (5.0 × 10^−7^–9.8 × 10^−7^). Scrambled *(C,A,G)_25_* and *(C,T,G)_25_* tracts were also tested, and median rates are as follows: *(C,A,G)_25_* wild type (*n* = 180) < 1.0 × 10^−8^, *msh3Δ* (*n* = 90) < 1.0 × 10^−8^, *msh6Δ* (*n* = 30) < 1.0 × 10^−8^, and *msh6(3MBD) msh3Δ* (*n* = 20) = 2.1 × 10^−7^ (1.6 × 10^−7^ to 3.5 × 10^−7^) and *(C,T,G)_25_*: wild type (*n* = 90) < 1.0 × 10^−8^, *msh3Δ* (*n* = 90) < 1.0 × 10^−8^, *msh6Δ* (*n* = 51) < 1.0 × 10^−8^, and *msh6(3MBD) msh3Δ* (*n* = 20) = 2.5 × 10^−7^ (1.5 × 10^−7^ to 3.6 × 10^−7^). *P*-values were calculated using Mann–Whitney test: **0.001 < *P* < 0.01; ****P < 0.0001. Please note the scale on the *y*-axis for CTG expansions is extended.

**Fig. 5.**
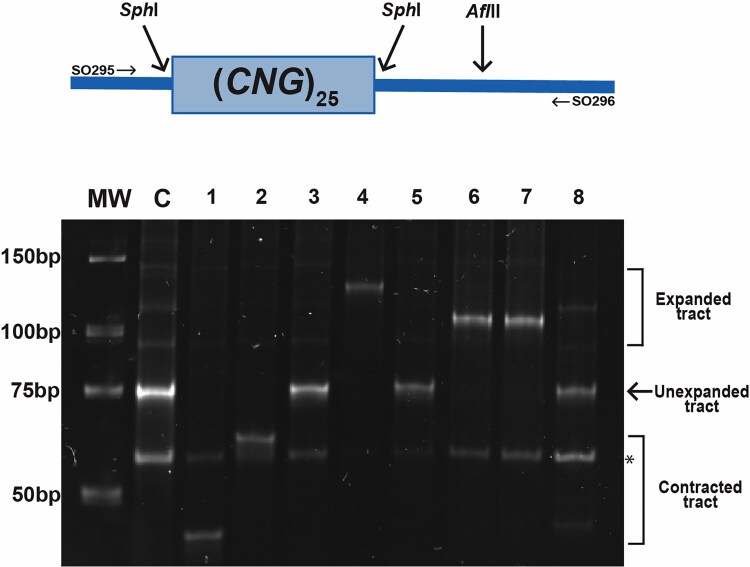
TNR tract length in reporters recovered from 5-FOA-resistant colonies. Top: Schematic of PCR product used to measure tract lengths of 5-FOA-resistant colonies. Amplification with SO295 and SO296 results in a 188 bp product. Digestion with *Sph*I cuts on either side of the tract, releasing the 75 bp tract. The remaining 73 bp product adjacent to the tract is digested with *Afl*II, allowing for visualization of the TNR tract. Bottom: Representative gel of digested TNR tracts amplified from 5-FOA-resistant colonies from wild type and *msh6(3MBD) msh3Δ*. These tracts include contracted (lanes 1 and 2), expanded (lanes 4, 6, and 7), and stable TNR tracts (lanes 3, 5, and 8). *denotes band that results from *Afl*II digestion.

We next sought to determine whether the Msh3 MBD was sufficient to promote expansions in vivo (Shell *et al*. 2007). We used an in vivo TNR expansion assay in *msh6(3MBD) msh3Δ* to determine the effect of this construct on *CAG* and *CTG* expansion rates. This assay, described previously (Miret *et al*. 1998; Williams *et al*. 2020), places a *URA3* reporter gene downstream of a promoter that encodes a (*CNG*)_25_ repeat tract. If an expansion of 4 or more repeats occurs in the tract (≥29 repeats), *URA3* will not be transcribed, leading to 5-FOA resistance (Williams and Surtees 2018). We previously demonstrated that *msh3Δ* reduces the expansion rate for (*CAG*)_25_ and (*CTG*)_25_ repeat tracts 5- and 30-fold, respectively, while the expansion rate was slightly increased in *msh6Δ* (Fig. 4) (Kantartzis *et al*. 2012). In *msh6(msh3MBD) msh3Δ*, the expansion rate for the (*CAG*)_25_ tract was very similar to that of the *msh3Δ*, while the *(CTG*)_25_ expansion rate was slightly lower than *msh3Δ* (Fig. 4). These results indicate that *msh6(3MBD)* does not complement an *msh3Δ*. Therefore, the Msh3 MBD is not sufficient to promote expansions and does not confer the capacity to promote TNR expansions on Msh6, despite the fact that Msh2-msh6(msh3MBD) is able to bind specifically to TNR structures. Mutations in *URA3* can also lead to 5-FOA resistance. Therefore, we determined the proportion of true expansions by amplifying the TNR tract from 5-FOA-resistant colonies and determining tract lengths by gel electrophoresis (Fig. 5) (Williams *et al*. 2020). In wild-type cells, 90% of *CAG* and >99% of *CTG* tracts exhibited true expansions. In *msh3Δ*, 63% of *CAG* and 91% of *CTG* tracts and, in *msh6Δ*, 55% of *CAG* and 90% of *CTG* tracts were bona fide TNR expansions (Kantartzis *et al*. 2012). In *msh6(3MBD) msh3Δ*, 7% of *CAG* tracts and 33% of *CTG* tracts exhibited true expansions (Table 6). The remaining tracts were either stable in length or exhibited tract contractions. We also observed a high rate of 5-FOA resistance in these strains with scrambled tracts, which do not expand (Fig. 4 legend), indicating that, in *msh6(3MBD) msh3Δ*, which has a high mutation rate (Fig. 3), mutations other than TNR tract expansions are leading to 5-FOA resistance. One possible source is mutations within *URA3* itself. We sequenced *URA3* from a subset of 5-FOA-resistant colonies associated with contracted (*CAG*) or stable (*CTG*) TNR tracts. For the contracted (*CAG*) tracts, 7 of 10 sustained a mutation within the coding sequence of *URA3*: H61Y, Y84 to STOP, and S154 to STOP each occurred twice, G255D occurred once. In contrast, for the stable (*CTG*) tracts, 0 of 17 sustained a mutation within the *URA3* coding or promoter region, and we infer that mutations elsewhere are leading to 5-FOA resistance eg (Armstrong *et al*. 2024).

**Table 6.** Proportion of true expansions in 5-FOA-resistant colonies.

		Permissive			Selective (5-FOA)		
Tract	Genotype	Expansion	Stable	Contraction	Expansion	Stable	Contraction
(*CAG*)_25_	*MSH6 MSH3*	0	61 (100%)	0	95 (90%)	11 (10%)	0
	*msh6(3MBD) msh3Δ*	0	10 (100%)	0	2 (7%)	8 (28%)	19 (65%)
	*msh6Δ*	3 (6%)	47 (94%)	0	27 (55%)	22 (45%)	0
	*msh3Δ*	0	53 (56%)	42 (44%)	44 (67%)	16 (24%)	6 (9%)
(*CTG*)_25_	*MSH6 MSH3*	0	68 (100%)	0	112 (100%)	0	0
	*msh6(3MBD) msh3Δ*	0	10 (100%)	0	13 (33%)	25 (65%)	1 (2%)
	*msh6Δ*	0	30 (100%)	0	26 (90%)	3 (10%)	0
	*msh3Δ*	0	25 (100%)	0	21 (91%)	2 (9%)	0
(*C*,*A*,*G*)_25_ scrambled	*MSH6 MSH3*	0	40 (100%)	0	0	40 (100%)	0
	*msh6(3MBD) msh3Δ*	0	20 (100%)	0	0	20 (100%)	0
(*C*,*T*,*G*)_25_ scrambled	*MSH6 MSH3*	0	60 (100%)	0	0	65 (100%)	0
	*msh6(3MBD) msh3Δ*	0	20 (100%)	0	0	20 (100%)	0

The source of 5-FOA resistance notwithstanding the false positive rate indicated that the calculated expansion rates for *msh6(3MBD) msh3Δ* are overestimates; a corrected rate for *msh6(3MBD) msh3Δ* TNR expansions would go from 2.5 × 10^−7^ to 1.8 × 10^−8^ (*CAG*) or from 6.6 × 10^−7^ to 2.2 × 10^−7^ (*CTG*). Thus, measuring TNR expansion rates with this assay becomes more complicated as the background mutation rate increases for a given genotype, increasing the probability of observing 5-FOA resistance without expansion. Nonetheless, our data suggest that the *CAG* and *CTG* expansion rates for the chimeric complex are ∼8- and ∼4-fold lower than in the absence of *MSH3*, respectively, with corrected expansion rates of 1.5 × 10^−7^ (*msh3Δ CAG*) and 9.7 × 10^−7^ (*msh3Δ CTG*).

## Discussion

We demonstrated that, in vitro, Msh2-msh6(3MBD) exhibited DNA structure-binding affinities for loop and TNR structures that were comparable to Msh2-Msh3, indicating that Msh2-msh6(3MBD) has acquired Msh3 DNA-binding properties and is able to recognize and bind TNR structures in vivo (Table 4). We also observed elevated affinities of Msh2-Msh6 for the TNR structures (Table 5), although Msh2-Msh6 does not promote expansions in vivo (Kantartzis *et al*. 2012). We also demonstrated that *msh6(3MBD) msh3Δ* is sufficient to allow some repair of Msh2-Msh3-specific DNA errors (in/dels), partially complementing the loss of *MSH3* in 4 nt. loop repair in vivo (Fig. 3). We hypothesize that differences in communication between the DNA-binding and ATPase domains of Msh2-Msh3 vs Msh2-Msh6 are reflected in this partial complementation. In contrast, this level of activity in *msh6(3MBD) msh3Δ* is not sufficient to promote MMR-mediated *CAG/CTG* expansions (Fig. 4). In fact, the true TNR expansion rates appear to be lower in *msh6(3MBD) msh3Δ* than in *msh3Δ*. Together, our data indicate that recognition and specific binding to the TNR structure by MSH complexes are not sufficient to promote TNR expansions. Thus, we propose that distinct Msh3-specific molecular requirements beyond Msh3 MBD are necessary for promoting TNR expansions, including DNA-mediated modulation of Msh2-Msh3 ATP-binding and hydrolysis and interactions with MLH complexes. Furthermore, Msh2-msh6(3MBD), a MSH complex that is able to bind MMR and TNR structures (Table 4) but not coordinate efficient repair (Figs. 3 and 4), appears to block background TNR expansions.

Msh2-Msh3 DNA binding to distinct structures is communicated to the ATPase domain through the connector domain, modulating its ATP binding, hydrolysis, and turnover activities to promote repair (Owen *et al*. 2005, 2009; Gupta *et al*. 2011; Lang *et al*. 2011; Kumar *et al*. 2014). ATP promotes Msh2-Msh3 dissociation from the DNA, promoting the recycling of Msh2-Msh3 (Surtees and Alani 2006; Brown *et al*. 2016). Different DNA structures modulate Msh2-Msh3 ATP binding and hydrolysis (Owen *et al*. 2005, 2009; Surtees and Alani 2006; Kumar *et al*. 2014). Critically, the Msh2-Msh3 ATP-binding domains are distinct from those of Msh2-Msh6, with regulated access to the Msh3 nucleotide-binding pocket (Gupta *et al*. 2011; Kumar *et al*. 2013). Altered regulation of ATP binding and/or hydrolysis disrupts Msh2-Msh3-mediated MMR, but not Msh2-Msh3's DSBR activity (Kumar *et al*. 2013). Therefore, Msh2-msh6(3MBD) may misregulate the ATPase domain through incorrect signal transduction after DNA binding and altered access to the nucleotide-binding pocket. Our previous data indicate that Msh2-msh6(3MBD) has a higher ATPase activity than Msh2-Msh3, more similar to Msh2-Msh6, while being stimulated by Msh2-Msh3-specific DNA substrates (Brown *et al*. 2016). One possibility is that this elevated ATPase activity increases the turnover of Msh2-msh6(3MBD), impairing both Msh2-Msh3-mediated MMR and TNR expansions, although apparently not to the same extent (Figs. 3 and 4) (Shell *et al*. 2007). This may be a result of distinct DNA structure-specific allosteric changes within the Msh complex. We propose that increased turnover precludes the Msh complex from targeting the DNA structures for either MMR or TNR expansion, leading to defects in both Msh2-Msh3-mediated pathways. Together, our data indicate that Msh2-Msh3's MMR activity is specifically required for promoting TNR expansions.

ATP-induced conformational changes are required for Msh2-Msh3's interaction with MLH complexes and stimulate their endonuclease activity. MutLα (yeast Mlh1-Pms1) is the primary Mlh complex in MMR, although MutLγ (yeast Mlh1-Mlh3) also plays a minor, largely Msh2-Msh3-specific, role in MMR. Both MutLα and MutLγ promote TNR expansions in vivo in mammalian systems in a Msh2-Msh3-specific manner (Pinto *et al*. 2013; Zhao *et al*. 2018; Hayward *et al*. 2020; Kadyrova *et al*. 2020; Miller *et al*. 2020; Roy *et al*. 2021; Lee *et al*. 2022), likely by nicking and promoting excision of the template leading to expansions (Pluciennik *et al*. 2013; Kadyrova *et al*. 2020). Our data support the hypothesis that this is not simply a result of altered DNA-binding specificity, but rather that Msh2-Msh3-specific Mlh interactions and activation are required for TNR expansions and are missing in Msh2-msh6(3MBD). We note that human Msh2-Msh3 interactions with MutLα are mediated through the PCNA interaction motif (PIP box) with the Msh3 N-terminal region (Pluciennik *et al*. 2013). In contrast, the Msh6 NTR or PIP box is not required for this interaction (Iyer *et al*. 2008); therefore, any Msh2-msh6(3MBD) interactions with MLH complexes are expected to be quite different from Msh2-Msh3-MLH interactions. We propose that Msh2-Msh3-mediated TNR expansions require the Msh2-Msh3-mediated MMR pathway to be fully functional and intact. This would include proper DNA binding and appropriate signal transduction for regulation of ATP binding/hydrolysis and subsequent Mlh interactions. This is consistent with Msh2-Msh3 playing an active, pathogenic role in promoting TNR expansions.

## Data Availability

All strains and plasmids are available upon request.
